# Sex-specific association of immunological markers in CS-delivered newborns with pre-pregnancy body mass index and gestational weight gain of mothers

**DOI:** 10.1038/s41598-025-85711-x

**Published:** 2025-01-24

**Authors:** Karolina Rak, Michaela Godyla-Jabłoński, Monika Bronkowska

**Affiliations:** 1 Department of Human Nutrition, Faculty of Biotechnology and Food Science, Wrocław University of Environmental and Life Sciences, Wrocław, 51-630 Poland; 2 Institute of Health Sciences, Collegium Salutis Humanae, University of Opole, Opole, 45-060 Poland

**Keywords:** Adaptive immunity, Neonatology, Nutrition, Immunology, Risk factors

## Abstract

Just as overweight and obesity may impair immunity, excessive body weight-related parameters of women in the pre-conception period and during pregnancy are possible detrimental factors for fetal programming of the immune system in their offspring. We investigated the relationship of pre-pregnancy body mass index (pBMI) and gestational weight gain (GWG) of mothers with the placental transport rate (PTR) of IgG antibodies and antineutrophil cytoplasmatic antibodies against lactoferrin (Lf-ANCA) and their concentration in umbilical cord blood serum (UCS), verifying the sex-specificity of this relationship. The examined group of this cross-sectional pilot study consisted of 101 pregnant women and their healthy CS-delivered newborn children. The concentration of antibodies in maternal serum (MS) and UCS were determined by ELISA method. PTR was assessed as a ratio of the concentration of antibodies in the UCS and MS. A significantly lower PTR of IgG and their concentration in the UCS were demonstrated in newborns of mothers with an excessive pBMI compared to those with pBMI < 25 and the association was more pronounced in male newborns. The lowest PTR of beneficial IgG and their concentration in the UCS as well as the highest PTR of detrimental Lf-ANCA and their concentration in UCS were observed in newborns born to mothers with co-occurrence of both an excessive pBMI and GWG. It seems that maternal preconception overweight and obesity along with an excessive GWG can be a predictor of unfavorable immune effects in fetuses. Further studies are needed to explain the role of maternal weight-related parameters in the development of immunological health of their offspring.

**Supplementary Information:**

The online version contains supplementary material available at 10.1038/s41598-025-85711-x.

## Introduction

Nutritional status in the pre-conception period and during pregnancy is particularly important, not only affecting maternal health but also prenatal development and the neonatal heath condition. As previously established, developmental and health-related patterns imprinted in early life have health consequences in later life^1–5^. Unfavorable conditions of the intrauterine environment trigger fetal programming, which allows the fetus to survive but also causes long-term adverse health effects, such as increased risk of diabetes mellitus, hypertension, atherosclerosis, obesity and other cardiometabolic diseases in adulthood^1,2,6–10^. While the issue of fetal programming of cardiometabolic diseases has been very accurately described in the literature^1,2,6–10^, the problem of fetal programming of the immune system and prenatal factors, including maternal nutritional status, that can affect the immunological defense potential of newborns remains poorly understood.

Development of the immune system begins in fetal life and maternal nutritional status is one of the key factors affecting this process both indirectly and directly^11–16^. The nutritional status of the mother may indirectly shape the developing immune system of the fetus, affecting:

1. the quantity and quality of maternal antibodies,
2. their availability for placental transport,
3. the size and morphology of the placenta and.
4. the vascular flow and efficiency of the placental transport of antibodies and nutrients essential for development of the fetal immune system^17^.

Moreover, the nutritional status of the mother directly affects the nutritional status of the fetus, which has a significant effect on its own developing immune system through:

1. regulation of the expression of genes (based on epigenetic modifications),
2. organogenesis of lymphoid organs and lymphogenesis, as well as.
3. regulation of the fetal hypothalamic-pituitary-adrenal axis, the activation of which results in an immunosuppressive effect^17^.

Excessive maternal weight-related parameters may be suspected of having a negative effect on fetal programming of the immunological system and its defensive capability in newborns as obesity – connected to chronic inflammation, oxidative stress and metabolic disorders^18–20^ – has been demonstrated to impair immunological functions related to both innate and acquired immunity, including reduced number of white blood cells, limited efficiency of phagocytosis and oxygen burst, weakened proliferation of lymphocytes and reduced production of antibodies^21,22^ that seems to be supported by study results demonstrating increased susceptibility to infection in obese people^23^.

Neonatal immune system, although currently considered as a “vigilance complex system” rather than immature structure^24,25^, is definitely functionally different compared to its adult counterpart. Specific immunity in newborns at birth is based primarily on maternal-derived IgG antibodies – the only class of antibodies crossing placental barrier^26^ and the key source of a highly precise specific immune response in a neonatal life. Although newborns are able to produce small amounts of IgA (responsible for the protection of mucous membranes) and IgM antibodies (involve in early immune reactions) and, as a result of breastfeeding after birth, obtain significant amounts of secretory IgA antibodies, they are unable to produce IgG antibodies which play a key role in a full-scale fight against pathogenic microorganism^27,28^. Moreover, neonatal non-specific immunity at birth is characterized by a down-regulated responsiveness and reactivity and anti-inflammatory phenotype^24,29,30^ caused by necessity of immunological tolerance to partially antigenetically different maternal organism^27,31^ and no significant contact with the external environment in the prenatal life^26,29,30^. Non-specific immunity in newborns, despite its decreased activity, nevertheless constitute the first line of defense, including the key role of neutrophils. These immune cells are able to release from their granules various antimicrobial proteins, including lactoferrin which presents an extraordinary broad spectrum of antibacterial, antivirus, antiparasitic, and antifungal properties as well as many immunomodulatory activities^30,32–34^. Insufficiency of lactoferrin has been shown to be linked to recurrent infections^35,36^, whereas supplementation with lactoferrin can reduce the risk of their occurrence^37–39^. Due to its indisputable role in the organism’s defense against infections and in regulation of immunological response, the presence of antineutrophil cytoplasmatic antibodies against lactoferrin (Lf-ANCA) in the blood should be considered as highly detrimental. In the literature, these auto-antibodies have been demonstrated to be linked to various autoimmune and inflammatory diseases^40–42^, however there is no data on Lf-ANCA antibodies in the obstetric context, including their placental transport to the fetus and factors that could affect it.

Identification of factors weakening the placental transport of essential IgG antibodies as well factors enhancing the placental transport of unfavorable Lf-ANCA auto-antibodies is of great importance. It may not only contribute in the recognition of at-risk newborns being especially susceptible to infections, but possibly even prevent immunologically adverse effects in newborns by attempting to alleviate or eliminate these factors. Some maternal factors limiting placental transport of IgG antibodies to the fetus have been described, such as malnutrition, diabetes, hyperglycaemia, chronic infections (including malaria and HIV) and hypergammaglobulinaemia^43^. However, maternal body weight-related parameters are very poorly examined in the context of their impact on the immune functions of the offspring. The current knowledge about Lf-ANCA auto-antibodies in this area is currently even poorer.

Although in obstetric studies, maternal obesity has been shown to be related to impaired functions of specific and non-specific immune cells in newborns^44–46^ as well as to an increased risk of infections in both neonatal period^47,48^ and adult life^49,50^, that has been also supported by the results from animal research^51,52^, to the best of our knowledge, there are no studies on the relationship of maternal body weight-related parameters with the placental transport rate of IgG antibodies and Lf-ANCA auto-antibodies and their concentration in cord blood.

We therefore aimed to investigate the relationship of maternal pre-pregnancy body mass index (pBMI) and gestational weight gain (GWG) with the placental transport rate (PTR) of IgG antibodies and Lf-ANCA auto-antibodies and their concentration in umbilical cord blood serum (UCS), verifying the sex-specificity of this relationship.

## Materials and methods

### Participants

A total of 101 healthy neonates (54 females, 47 males) born in the Obstetrics and Gynaecology Ward of the Provincial Specialist Hospital in Wroclaw Research and Development Centre (Poland) between April 2016 and December 2016 and their mothers were enrolled in the cross-sectional pilot study. All examined neonates were full term (≥ 37th week Hbd) with birth weight ≥ 2500 g in order to exclude potential negative influence of prematurity and low birth weight (LBW) on the immunological markers^53,54^. All examined women were healthy and declared no tobacco or alcohol use throughout the pregnancy and had uncomplicated pregnancies terminated with caesarean section (CS) due to obstetric (previous CS), ophthalmological (advanced eye defect) or other indications (e.g. tokophobia). Women with natural labours and multiple pregnancies were not enrolled in the study due to difficulties in obtaining their informed consent due to the dynamic course of natural childbirth in patients arriving at the hospital during labor or due to the commonly declared LBW predicted by USG assay in newborns from multiple pregnancies.

### Determination of pre-pregnancy BMI and gestational weight gain

Data on body height, pre-pregnancy body weight and body weight just before CS were received from the personal pregnancy record books of the examined women. The pBMI was calculated according to the formula pBMI = pre-pregnancy body weight [kg] / body height [m^2^]. In the first part of analyses, this parameter was considered as a continuous variable, while in the second part of analyses pBMI was categorized into < 25 [kg/m^2^] and ≥ 25 [kg/m^2^]. GWG was calculated as the difference between the body weight just before CS and body weight in the pre-conception period. In the first part of analyses, this parameter was considered as a continuous variable, while in the second part of analyses GWG was categorized into “not excessive” (adequate or insufficient) and excessive based on the recommendations of the Institute of Medicine and National Research Council^55^ (see Table 1).

**Table 1:** Recommended weight gain in a single pregnancy depending on pre-pregnancy BMI.

BMI status	pBMI [kg/m^2^]	GWG [kg]
Undernutrition	< 18,5	12,5–18,0
Normal body weight	18,5–24,9	11,5–16,0
Overweight	25,0–29,9	7,0–11,5
Obesity	≥ 30	5,0–9,0

*pBMI* pre-pregnancy BMI, *GWG* gestational weight gain.

### Determination of the concentration of IgG and Lf-ANCA antibodies in maternal and cord blood serum

MS samples were collected before planned CS (no labour), whereas UCS samples were collected during CS. Blood samples after clotting were centrifuged for 15 min at 2000 rpm. Serum samples were removed and stored at −80 °C for a maximum of 12 months (depending of the sample)until analysis^56,57^.The concentration of IgG and Lf-ANCA antibodies in the MS and UCS were measured in duplicate by enzyme-linked immunosorbent assay (*Human IgG Total Ready-SET-Go! ELISA*, eBioscience, Bender MedSystem GmbH, Vienna, Austria and *Lactoferrin Ab ELISA*, Demeditec Diagnostics GmbH, Kiel, Germany, respectively) according to the protocols recommended by the manufacturers and included in the operating instructions. ELISA test protocols provided internal quality control of measurements by requiring preparation of standard curves, positive and negative controls in duplicate. Absorption was measured with an Epoch plate reader (BioTek Instruments, USA). Concentrations of IgG antibodies were expressed as mg/dl, while concentrations of Lf-ANCA antibodies – as U/ml.

The PTR of IgG antibodies was determined as the ratio of the concentration of IgG in the UCS and MS (UCS/MS IgG). Similarly, the PTR of Lf-ANCA antibodies was determined as the ratio of the concentration of Lf-ANCA in the UCS and MS (UCS/MS Lf-ANCA).

### Statistical analysis

Statistical analysis was performed using Statistica software, version 13.0. Non-parametric tests were used as data did not meet the conditions needed for parametric tests (non-normal distributions, the lack of homogeneity of variance, small size of examined subgroups). Spearman’s correlation was applied to estimate the association between continuous variables. The Mann-Whitney test was used to compare differences between two groups of independent variables, while the Kruskal –Wallis test was employed to compare differences between three groups of independent variables and NIR as a post-hoc test. The surfaces presenting three-dimensional relationships between variables were created by the distance-weighted least squares smoothing. For all analyses, *p* ≤ 0.05 was considered significant^58^. In the Results section below, size of subgroup (*N*), mean, standard deviation (SD), median (Me), interquantile range (IQR), range (Min – Max), Mann-Whitney *Z* value (MW-Z), Kruskal-Wallis *H* value (KW-H), strength of Spearman’s correlation (rho) and *p* value are presented.

## Results

### Participants characteristics

The baseline characteristics of mothers and newborns are presented in Table 2. The examined women were aged from 19 to 45 years, average 32.89 ± 4.67 years. The mean pBMI was 23.21 ± 4.30 kg/m^2^, with 28.7% being overweight/obese (i.e. BMI ≥ 25 kg/m^2^). An almost equal proportion of an adequate and excessive GWG was observed (47.5% vs. 44.6%) with only 8 women with an insufficient GWG. The number of female and male newborns was similar (54 vs. 47, respectively) with no significantly statistical sex-dependent differences in gestational age, birth weight, the concentration of IgG and Lf-ANCA antibodies in the UCS and their PTR.

**Table 2:** Characteristics of mothers and newborns (*N* = 101).

	*N*	Mean	SD	Median	IQR	Range	*p*-value
Maternal characteristics							
Maternal age [years]	101	32.89	4.67	33.00	30.00–35.00	19.00–45.00	
Pre-pregnancy BMI [kg/m^2^]^1^	101	23.21	4.30	22.14	20.07–25.31	16.72–40.12	
Gestational weight gain [kg]^2^	101	14.88	4.67	14.00	12.00–18.00	5.00–27.50	
MS IgG [mg/dl]	77	448.45	160.60	465.00	348.00–547.00	90.00–946.00	
MS Lf-ANCA [U/ml]	101	61.17	63.49	49.93	20.00–81.10	0.00–416.90	
Neonatal characteristics^3^							
Gestational age [weeks]	101	38.78	0.87	39.00	38.00–39.00	37.00–41.00	
♀	54	38.87	0.92	39.00	38.00–39.00	37.00–41.00	0.234
♂	47	38.67	0.78	39.00	38.00–39.00	37.00–41.00	
Birth weight [g]	101	3468.25	453.46	3400.00	3140.00–3730.00	2490.00–4980.00	
♀	54	3447.09	430.85	3380.00	3130.00–3710.00	2580.00–4980.00	0.641
♂	47	3492.50	481.51	3470.00	3160.00–3750.00	2490.00–4660.00	
UCS IgG [mg/dl]	77	473.25	173.55	474.50	360.50–559.50	78.00–1096.00	
♀	41	457.10	184.31	462.00	319.00–548.00	78.00–1096.00	0.158
♂	36	491.10	161.38	497.00	418.00–569.00	104.00–901.00	
UCS/MS IgG^3^	77	1.12	0.42	1.03	0.87–1.21	0.44–2.80	
♀	41	1.13	0.47	1.00	0.87–1.21	0.44–2.80	0.607
♂	36	1.11	0.35	1.03	0.95–1.25	0.68–2.74	
UCS Lf-ANCA [U/ml]	101	28.92	38.00	12.80	0.00–40.30	0.00–163.10	
♀	54	27.92	37.16	13.90	0.00–40.60	0.00–155.90	0.711
♂	47	30.07	39.32	10.40	0.00–40.30	0.00–163.10	
UCS/MS Lf-ANCA	101	0.63	0.61	0.59	0.03–1.00	0.00–3.70	
♀	54	0.57	0.51	0.56	0.00–1.00	0.00–1.90	0.443
♂	47	0.70	0.70	0.67	0.11–1.00	0.00–3.70	

*MS* maternal serum, *UCS* umbilical cord serum, *IgG* antibodies in G class, ♀ female newborns (*N* = 54), ♂ male newborns (*N* = 47), IQR – interquartile range.

^1^pBMI < 18.5 *N* = 6 (5.9%), 18.5–24.9 *N* = 66 (65.4%), 25–29.9 *N* = 19 (18.8%), 30–34.99 *N* = 8 (7.9%), 35–39.99 *N* = 1 (1.0%), 40–44.99 *N* = 1 (1.0%).

^2^GWG – insufficient *N* = 8 (7.9%), adequate *N* = 48 (47.5%), excessive *N* = 45 (44.6%);

^3^Sex of newborns: female *N* = 54 (53.5%), male *N* = 47 (46.5%).

### The relationship of IgG in the MS with IgG in the UCS and PTR of IgG

**Fig. 1:**
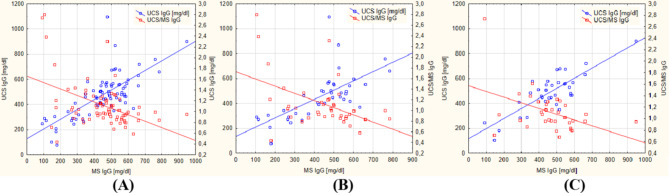
The scatterplots of concentration of IgG antibodies in umbilical cord blood serum (UCS IgG) and the placental transport rate (PTR) of IgG antibodies – expressed as the ratio of the concentration of IgG in the UCS and MS (UCS/MS IgG) – in relation to the concentration of IgG antibodies in maternal serum (MS IgG) in all newborns (regardless of sex) (**A**), female newborns (**B**) and male newborns (**C**).

Regardless of sex, a strong positive correlation between the concentration of IgG antibodies in the MS and their concentration in the UCS was observed (all newborns: rho = 0.726, *p* < 0.000; females: rho = 0.661, *p* < 0.000; males: rho = 0.820, *p* < 0.000), while a moderate negative correlation between the concentration of IgG antibodies in the MS and their PTR was demonstrated (all newborns: rho = − 0.456, *p* < 0.000; females: rho = − 0.470, *p* = 0.002; males: rho = − 0.443, *p* = 0.007) (Fig. 1).

### The relationship of Lf-ANCA in the MS with Lf-ANCA in the UCS and PTR of Lf-ANCA

**Fig. 2:**
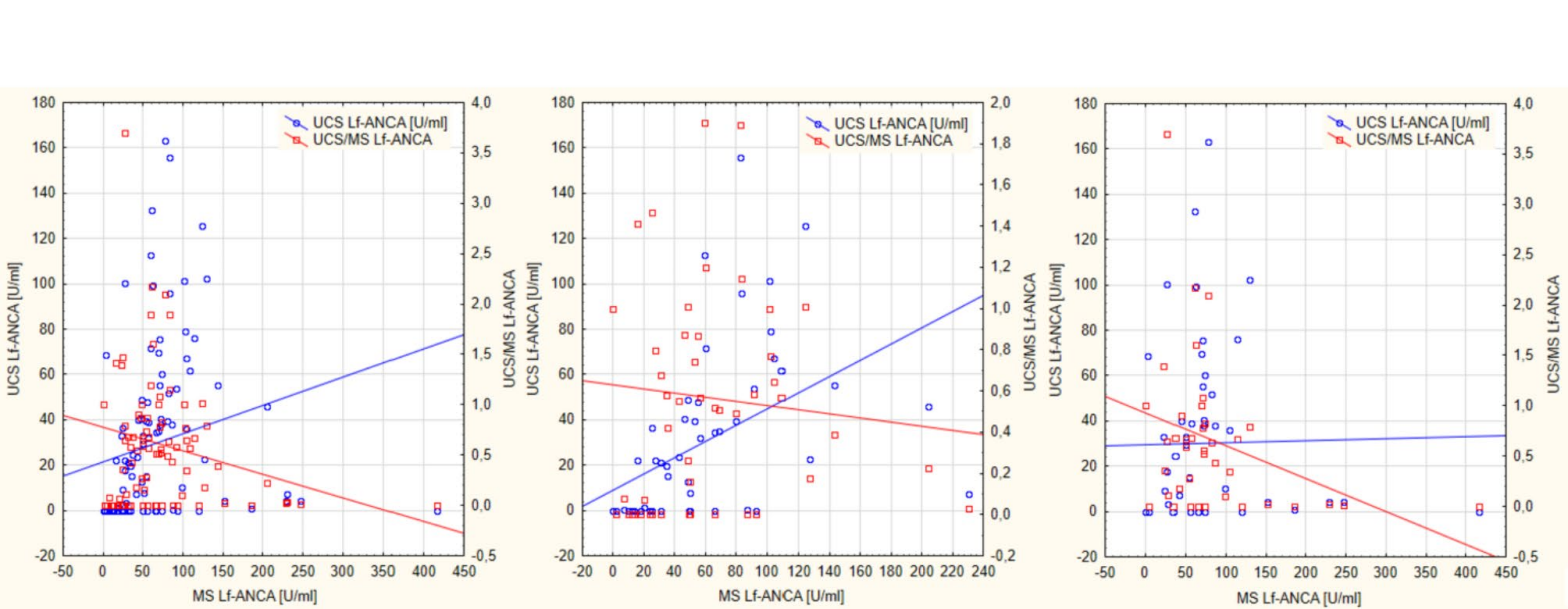
The scatterplots of concentration of anti-lactoferrin antibodies in umbilical cord blood serum (UCS Lf-ANCA) and the placental transport rate (PTR) of Lf-ANCA antibodies – expressed as the ratio of the concentration of Lf-ANCA in the UCS and MS (UCS/MS Lf-ANCA) – in relation to the concentration of Lf-ANCA antibodies in maternal serum (MS MS Lf-ANCA) in all newborns (regardless of sex) (**A**), female newborns (**B**) and male newborns (**C**).

Regardless of sex, a moderate positive correlation between the concentration of Lf-ANCA auto-antibodies in the MS and their concentration in the UCS was observed (all newborns: rho = 0.578, *p* < 0.000; females: rho = 0.710, *p* < 0.000; males: rho = 0.397, *p* = 0.006). A poor negative correlation between the concentration of Lf-ANCA auto-antibodies in the MS and their PTR was demonstrated in all and male newborns (rho = − 0.253, *p* = 0.010 and rho = − 0.470, *p* = 0.002; males: rho = − 0.475, *p* = 0.001, respectively) was demonstrated, while in female newborns no statistically significant correlation between these parameters was shown (Fig. 2).

### The concentration of examined antibodies (IgG and Lf-ANCA) in the MS and UCS and their PTR in relation to pre-pregnancy BMI and gestational weight gain

**Fig. 3:**
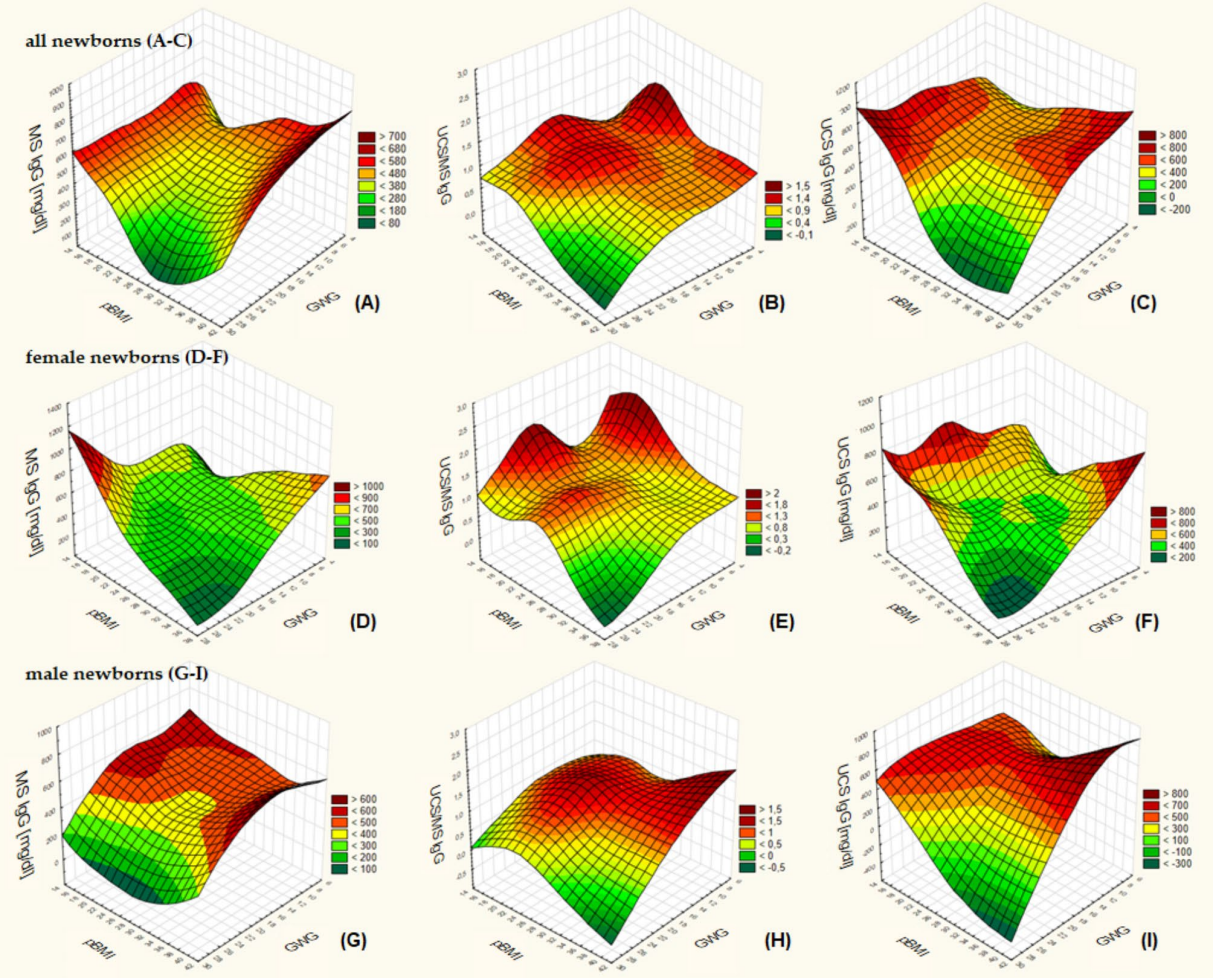
The surface plots of the concentration of IgG antibodies in maternal serum (MS IgG) and in cord blood serum of newborns (UCS IgG) and the placental transport rate (PTR) of IgG antibodies – expressed as the ratio of the concentration of IgG in the UCS and the MS (UCS/MS IgG) in relation to pre-pregnancy BMI (pBMI) and gestational weight gain (GWG). These relationships concern all newborns (regardless of sex) (**A**–**C**) and separately female newborns (**D**–**F**) and male newborns (**G**–**I**). The surfaces were created using the distance-weighted least squares smoothing.

The relationships of the concentration of IgG antibodies in the MS and UCS and PTR of maternal IgG antibodies with pBMI and GWG are presented on the Fig. 3 (A-I). In all newborns, regardless of sex, born to mothers with pBMI indicating overweigh or obesity (≥ 25) and simultaneously with a high GWG, the lowest PTR of maternal IgG antibodies (Fig. 3B, E, H) and their concentration in the UCS were demonstrated (Fig. 3C, F, I). Similar relationship, although not so pronounced, was observed for the concentration of IgG antibodies in the MS (Fig. 3A, D, G). The highest PTR of maternal IgG antibodies and their concentration in the UCS were shown in newborns born to mothers with an excessive pBMI along with a low GWG, an insufficient pBMI along with a high GWG, and a normal pBMI along with a moderate GWG (Fig. 3B-C, E-F, H-I). Similar relationship was shown for the concentration of IgG antibodies in the MS of examined women (Fig. 3A). However, sex-differences were observed. In mothers of female newborns, the highest concentration of IgG antibodies in the MS was strictly related to the co-occurrence of an insufficient or lower limit of normal pBMI (< 20) with a high GWG (> 22 kg) or pBMI indicating obesity with a very low GWG (< 6 kg) (Fig. 3D), whereas in mothers of male newborns the highest concentration of IgG antibodies in the MS was related to a low and moderate GWG, irrespective of pBMI (Fig. 3G).

**Fig. 4:**
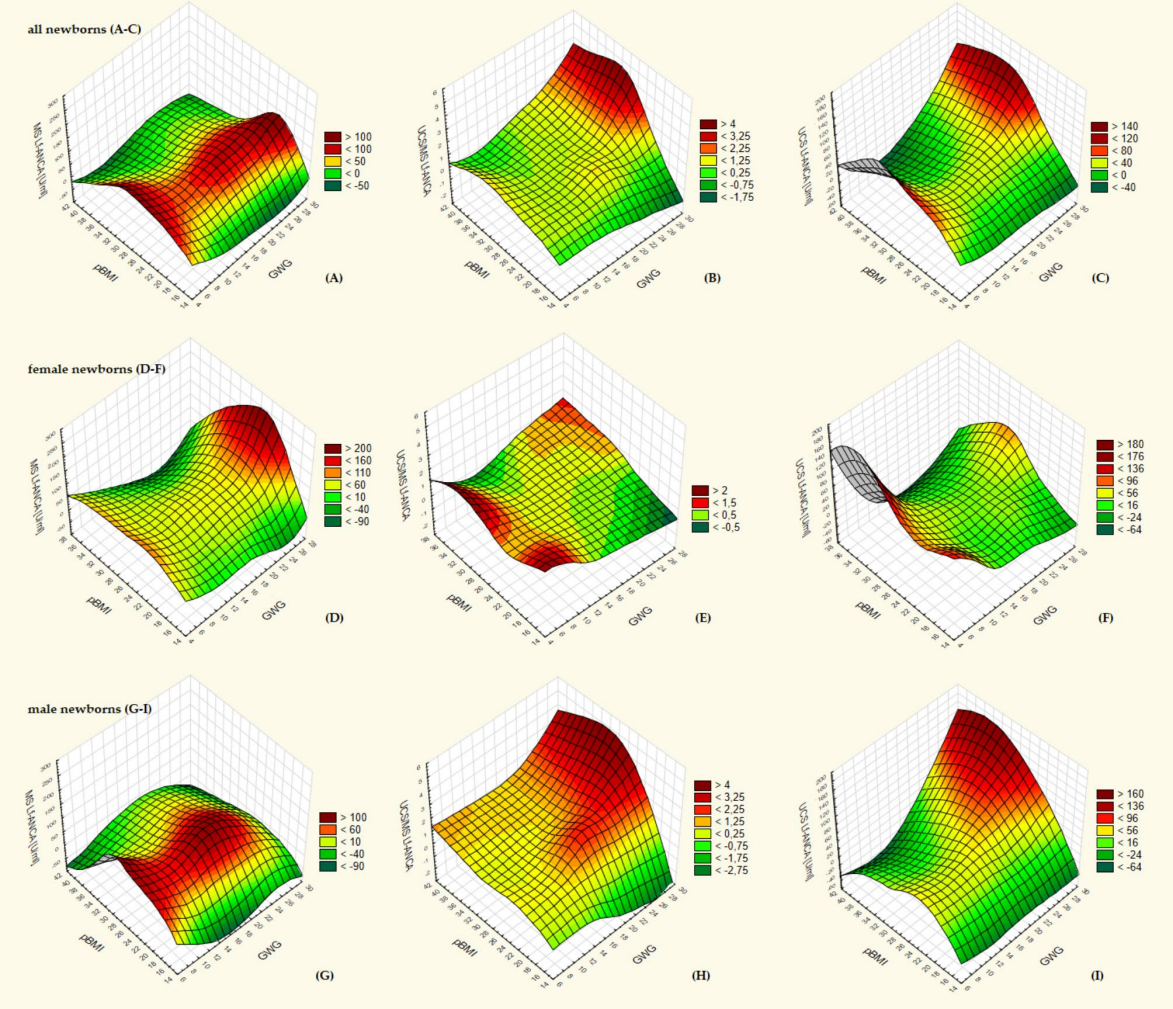
The surface plots of the concentration of anti-lactoferrin antibodies in maternal serum (MS Lf-ANCA) (**A**,**D**,**G**) and in cord blood serum of newborns (UCS Lf-ANCA) (**C**,**F**,**I**) and the placental transport rate (PTR) of Lf-ANCA antibodies – expressed as the ratio of the concentration of Lf-ANCA in the UCS and the MS (UCS/MS Lf-ANCA) (**B**,**E**,**H**) in relation to pre-pregnancy BMI (pBMI) and gestational weight gain (GWG). These relationships concern all newborns (regardless of sex) (**A**–**C**) and separately female newborns (**D**–**F**) and male newborns (**G**–**I**). The surfaces were created using the distance-weighted least squares smoothing.

The relationships of the concentration of Lf-ANCA antibodies in the MS and UCS and PTR of maternal Lf-ANCA antibodies with pBMI and GWG are presented on the Fig. 4 (A-I). The highest PTR of maternal Lf-ANCA antibodies and their concentration in the UCS were demonstrated in all newborns (Fig. 4B-C) and male newborns (Fig. 4H-I) born to mothers with pBMI indicating overweigh or obesity (≥ 25) and simultaneously with a high GWG (> 22 kg), whereas in female newborns (Fig. 4E-F) the highest PTR as well as the highest concentration in the UCS of Lf-ANCA antibodies were shown in those born to mothers with low GWG (< 12 kg), irrespective of pBMI. Interestingly, the highest concentration of these auto-antibodies were observed in the MS of women with normal or excessive pBMI, irrespective of GWG, gave birth to all (Fig. 4A) and male newborns (Fig. 4G), while in mothers of female newborns (Fig. 4D), the highest MS Lf-ANCA was related to normal and excessive pBMI along with a high GWG (> 25 kg). The lowest concentration of Lf-ANCA antibodies in the MS, regardless of sex, was related to undernutrition (pBMI < 18.5) as well as obesity II◦ (pBMI ≥ 35), irrespective to GWG (Fig. 4A, D, G). The lowest PTR of Lf-ANCA and their concentration in the UCS were not so clearly related to pBMI and GWG (Fig. 4B-C, E-F, H-I).

### The concentration of examined antibodies (IgG and Lf-ANCA) in the MS and UCS and their PTR in relation to categorized pre-pregnancy BMI and gestational weight gain

Analyses were conducted in order to present the concentration of IgG antibodies in the MS (Table 3) and UCS (Table 4), as well as the PTR of IgG antibodies (Table 5) in all newborns, female newborns and male newborns in relation to categorized weight-related parameters – pBMI categorized into < 25 and ≥ 25 as well as GWG categorized into “not excessive” and excessive. Women with insufficient pBMI were included in the category of pBMI < 25 (*N* = 6). Women with insufficient GWG were included in the category of “not excessive” (*N* = 8). No statistically significant relationship between examined parameters and the concentration of IgG in the MS was observed (Table 3). However, a significantly lower PTR of IgG antibodies was demonstrated in all newborns and male newborns of mothers with an excessive pBMI compared to those of mothers with pBMI < 25 (Table 5), while a significantly lower concentration of IgG antibodies in the UCS was observed only in male newborns born to mothers with an excessive pBMI (≥ 25) compared to those born to mothers with pBMI < 25. No significant relationship was shown between GWG and the concentration of IgG in the MS and UCS or their PTR.

**Table 3:** The concentration of IgG antibodies in maternal serum (MS IgG) and in blood serum of mothers of female newborns (♀) and male newborns (♂) in relation to pre-pregnancy BMI (pBMI) and gestational weight gain (GWG).

Examined parameters	MS IgG [mg/dl]			MW-Z	*p*	MS IgG [mg/dl] ♀			MW-Z	*p*	MS IgG [mg/dl] ♂			MW-Z	*p*
	*N*	Me	IQR			*N*	Me	IQR			*N*	Me	IQR		
pBMI [kg/m^2^]															
< 25^1^	57	470.00	370.00–535.00	− 0.589	0.556	31	473.00	346.00–524.00	− 0.852	0.394	26	476.50	394.00–547.00	0.071	0.944
≥ 25	20	505.00	332.00–579.00			10	570.00	146.00–679.50			10	475.50	435.00–515.00		
GWG [kg]															
Nxcessive^2^	43	486.00	416.00–551.00	1.462	0.144	24	490.00	428.00–555.00	1.643	0.100	19	470.00	410.00–547.00	0.222	0.824
Excessive	34	440.00	306.00–530.00			17	340.50	267.50–514.00			17	454.00	394.00–547.00		

*IgG* the concentration of IgG antibodies, *MS* maternal serum, *pBMI* pre-pregnancy body mass index, *GWG* gestational weight gain, *Me* median, *IQR* interquantile range, *MW-Z* the value of Mann-Whitney test, ♀ mothers of female newborns, ♂ mothers of male newborns, ^1^ pBMI < 25 – adequate (*N* = 51) + insufficient (*N* = 6), ^2^ not excessive GWG – adequate (*N* = 35) + insufficient (*N* = 8), * statistical significance *p* < 0.05.

**Table 4:** The concentration of IgG antibodies in umbilical cord blood serum (UCS IgG) of all newborns, female newborns (♀) and male newborns (♂) in relation to pre-pregnancy BMI (pBMI) and gestational weight gain (GWG) in examined women.

Examined parameters	UCS IgG [mg/dl]			MW-Z	*p*	UCS IgG [mg/dl] ♀			MW-Z	*p*	UCS IgG [mg/dl] ♂			MW-Z	*p*
	*N*	Me	IQR			*N*	Me	IQR			*N*	Me	IQR		
pBMI [kg/m^2^]															
< 25^1^	57	486.00	406.00–564.00	1.571	0.116	31	458.00	357.50–525.50	0.546	0.585	26	**547.00**	460.00–581.00	2.084	0.037*
≥ 25	20	431.50	272.00–542.00			10	394.00	267.00–597.00			10	**431.50**	319.00–488.00		
GWG [kg]															
Not excessive^2^	43	484.00	406.00–579.00	1.115	0.265	24	480.00	403.00–552.00	1.448	0.148	19	507.00	449.00–581.00	0.408	0.683
Excessive	34	464.00	289.00–545.50			17	332.50	272.00–503.00			17	497.50	418.00–568.00		

*IgG* the concentration of IgG antibodies, *UCS* umbilical cord blood serum, *pBMI* pre-pregnancy body mass index, *GWG* gestational weight gain, *Me* median, *IQR* interquantile range, *MW-Z* the value of Mann-Whitney test, ♀ female newborns, ♂ male newborns, ^1^pBMI < 25 – adequate (*N* = 51) + insufficient (*N* = 6), ^2^not excessive GWG – adequate (*N* = 35) + insufficient (*N* = 8) * statistical significance *p* < 0.05.

**Table 5:** The placental transfer rate of IgG antibodies (UCS/MS IgG) in all newborns, female newborns (♀) and male newborns (♂) in relation to pre-pregnancy BMI (pBMI) and gestational weight gain (GWG) in examined women.

Examined parameters	UCS/MS IgG			MW-Z	*p*	UCS/MS IgG ♀			MW-Z	*p*	UCS/MS IgG ♂			MW-Z	*p*
	*N*	Me	IQR			*N*	Me	IQR			*N*	Me	IQR		
pBMI [kg/m^2^]															
< 25^1^	57	**1.08**	0.95–1.24	2.267	0.023*	31	1.04	0.88–1.21	0.324	0.746	26	**1.10**	0.97–1.30	2.773	0.006*
≥ 25	20	**0.94**	0.80–1.08			10	1.00	0.86–1.08			10	**0.84**	0.79–0.98		
GWG [kg]															
Not excessive^2^	43	1.00	0.89–1.16	-0.587	0.558	24	0.95	0.85–1.18	-1.283	0.199	19	1.08	0.96–1.16	0.444	0.657
Excessive	34	1.04	0.89–1.25			17	1.05	0.94–1.22			17	0.98	0.86–1.25		

*IgG* the concentration of IgG antibodies, *UCS* umbilical cord blood serum, *MS* maternal serum, *pBMI* pre-pregnancy body mass index, *GWG* gestational weight gain, *Me* median, *IQR* interquantile range, *MW-Z* the value of Mann-Whitney test, ♀ female newborns, ♂ male newborns, ^1^pBMI < 25 – adequate (*N* = 51) + insufficient (*N* = 6), ^2^ not excessive GWG – adequate (*N* = 35) + insufficient (*N* = 8), * statistical significance *p* < 0.05.

Analogical analyses were conducted for Lf-ANCA (Tables 6, 7 and 8), however no statistically significant relationship between examined parameters were observed.

**Table 6:** The concentration of Lf-ANCA antibodies in maternal serum (MS Lf-ANCA) and in blood serum of mothers of female newborns (♀) and male newborns (♂) in relation to pre-pregnancy BMI (pBMI) and gestational weight gain (GWG).

Examined parameters	MS Lf-ANCA [U/ml]			MW-Z	*p*	MS Lf-ANCA [U/ml]♀			MW-Z	*p*	MS Lf-ANCA [U/ml]♂			MW-Z	*p*
	*N*	Me	IQR			*N*	Me	IQR			*N*	Me	IQR		
pBMI [kg/m^2^]															
< 25^1^	74	53.00	23.50–86.20	1.056	0.291	42	48.95	18.15–83.80	0.499	0.618	32	61.50	29.38–92.60	1.083	0.277
≥ 25	27	38.10	11.57–72.45			12	30.23	11.58–75.60			15	43.40	13.55–72.45		
GWG [kg]															
Not excessive^2^	58	49.40	20.00–77.90	− 0.560	0.575	32	46.50	15.70–65.79	− 1.211	0.226	26	58.90	27.40–81.10	0.528	0.598
Excessive	43	54.40	17.73–96.65			22	55.85	23.50–101.50			21	51.45	0.00–72.70		

*Lf-ANCA* the concentration of Lf-ANCA antibodies, *MS* maternal serum, *pBMI* pre-pregnancy body mass index, *GWG* gestational weight gain, *Me* median, *IQR* interquantile range, *MW-Z* the value of Mann-Whitney test, ♀ mothers of female newborns, ♂ mothers of male newborns, ^1^pBMI < 25 – adequate (*N* = 68) + insufficient (*N* = 6), ^2^not excessive GWG – adequate (*N* = 48) + insufficient (*N* = 10), * statistical significance *p* < 0.05.

**Table 7:** The placental transfer rate of Lf-ANCA antibodies (UCS/MS Lf-ANCA) in all newborns, female newborns (♀) and male newborns (♂) in relation to pre-pregnancy BMI (pBMI) and gestational weight gain (GWG) in examined women.

Examined parameters	UCS/MS Lf-ANCA			MW-Z	*p*	UCS/MS Lf-ANCA ♀			MW-Z	*p*	UCS/MS Lf-ANCA ♂			MW-Z	*p*
	*N*	Me	IQR			*N*	Me	IQR			*N*	Me	IQR		
pBMI [kg/m^2^]															
< 25^1^	74	0.58	0.02–1.00	− 0.994	0.320	42	0.56	0.00–1.00	− 0.510	0.610	32	0.615	0.02–1.00	− 0.764	0.445
≥ 25	27	0.70	0.18–1.00			12	0.54	0.19–1.00			15	0.68	0.18–1.00		
GWG [kg]															
Not excessive^2^	58	0.64	0.08–1.00	0.656	0.512	32	0.66	0.07–1.00	1.226	0.221	26	0.64	0.11–0.91	− 0.503	0.615
Excessive	43	0.56	0.02–1.00			22	0.46	0.00–0.77			21	0.67	0.11–1.00		

*Lf-ANCA* the concentration of Lf-ANCA antibodies, *UCS* umbilical cord blood serum, *MS* maternal serum, *pBMI* pre-pregnancy body mass index, *GWG* gestational weight gain, *Me* median, *IQR* interquantile range, *MW-Z* the value of Mann-Whitney test, ♀ female newborns, ♂ male newborns. ^1^pBMI < 25 – adequate (*N* = 68) + insufficient (*N* = 6), ^2^not excessive GWG – adequate (*N* = 48) + insufficient (*N* = 10)* statistical significance *p* < 0.05.

**Table 8:** The concentration of Lf-ANCA antibodies in umbilical cord blood serum (UCS Lf-ANCA) of all newborns, female newborns (♀) and male newborns (♂) in relation to pre-pregnancy BMI (pBMI) and gestational weight gain (GWG) in examined women.

Examined parameters	UCS Lf-ANCA [mg/dl]			MW-Z	*p*	UCS Lf-ANCA [mg/dl] ♀			MW-Z	*p*	UCS Lf-ANCA ♂			MW-Z	*p*
	*N*	Me	IQR			*N*	Me	IQR			*N*	Me	IQR		
pBMI [kg/m^2^]															
< 25^1^	74	11.60	0.00–40.30	− 0.138	0.890	42	10.38	0.00–40.60	− 0.218	0.827	32	12.80	0.00–40.20	0.046	0.964
≥ 25	27	15.00	0.00–55.50			12	23.50	0.00–46.05			15	7.40	0.00–60.20		
GWG [kg]															
Not excessive^2^	58	19.35	0.00–40.10	0.446	0.655	32	10.38	0.00–39.90	− 0.290	0.771	26	27.10	0.00–40.10	0.984	0.324
Excessive	43	7.00	0.00–47.80			22	17.40	0.00–47.80			21	4.10	0.00–40.30		

*Lf-ANCA* the concentration of Lf-ANCA antibodies, *UCS* umbilical cord blood serum, *pBMI* pre-pregnancy body mass index, *GWG* gestational weight gain, *Me* median, *IQR* interquantile range, *MW-Z* the value of Mann-Whitney test, ♀ female newborns, ♂ male newborns. ^1^pBMI < 25 – adequate (*N* = 68) + insufficient (*N* = 6), ^2^ not excessive GWG – adequate (*N* = 48) + insufficient (*N* = 10), *statistical significance *p* < 0.05.

### The concentration of examined antibodies (IgG and Lf-ANCA) in the MS and UCS and their PTR in relation to mixed categories of pre-pregnancy BMI and gestational weight gain

**Fig. 5:**
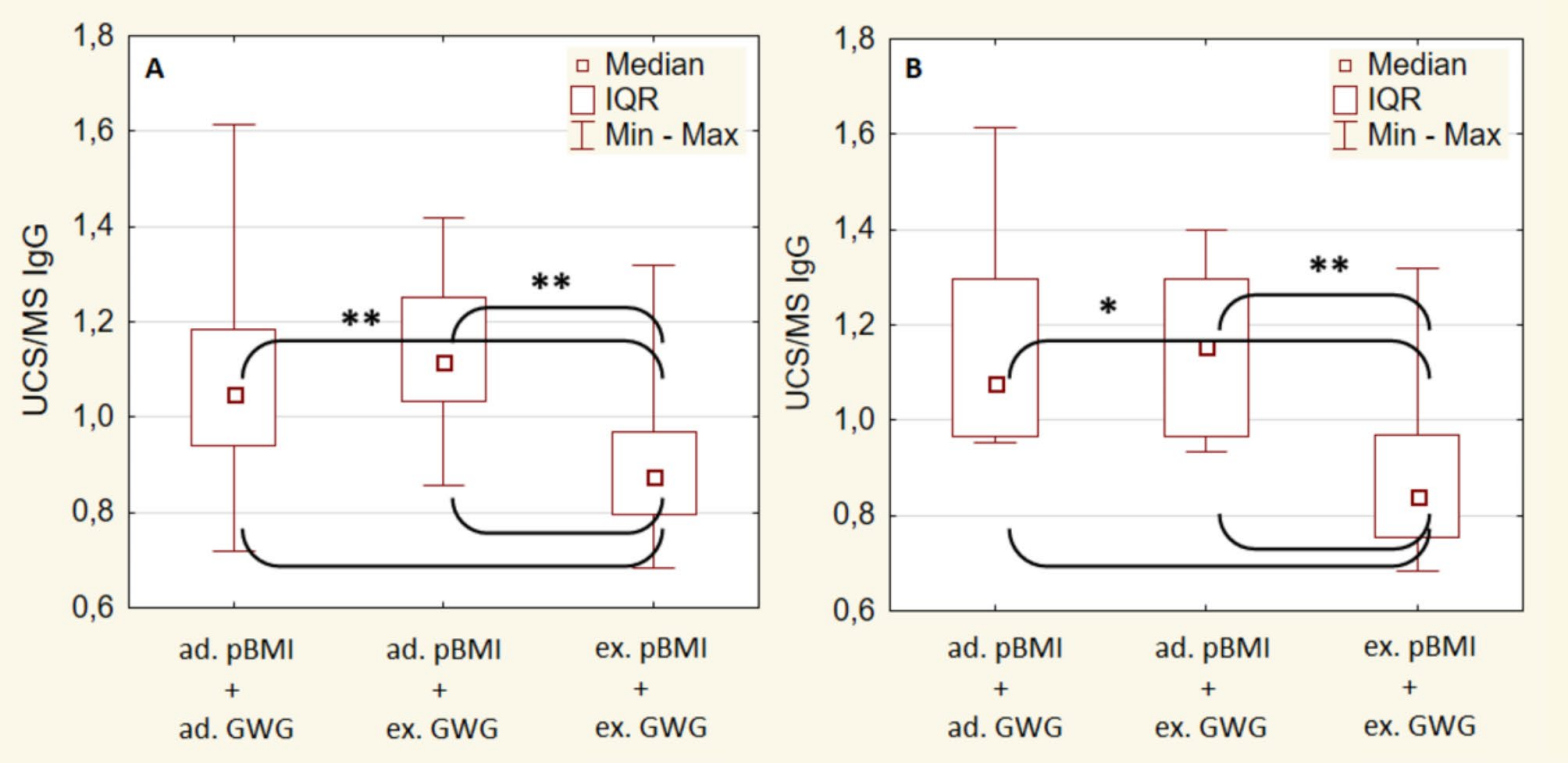
The placental transport rate (PTR) of IgG antibodies expressed as the ratio of the concentration of IgG in the UCS and the MS (UCS/MS IgG) in all newborns (**A**) and male newborns (**B**) in relation to pre-pregnancy BMI (pBMI) and gestational weight gain (GWG). Kruskal-Wallis test and post-hoc NIR test were used. *ad.* adequate, *ex.* excessive, * *p* < 0.05, ** *p* < 0.01.

The concentration of IgG antibodies and Lf-ANCA auto-antibodies in the MS and UCS and their PTR were examined in relation to 3 categories of pBMI and GWG: (1) adequate pBMI and adequate GWG, (2) adequate pBMI and excessive GWG, and (3) excessive pBMI and excessive GWG. There were no mothers with excessive pBMI and adequate GWG. Analyses were conducted for all newborns as well as for female and male newborns separately. The lowest PTR of IgG antibodies was demonstrated in all newborns (Fig. 5A) and male newborns (Fig. 5B) born to mother characterized with both excessive pBMI and GWG compared to those born to mothers with adequate pBMI and adequate or excessive GWG. No more statistically significant relationships were found.

## Discussion

In the course of this pilot research, we investigated maternal weight-related parameters such as pre-pregnancy body mass index (pBMI) and gestational weight gain (GWG) with the placental transport rate (PTR) of IgG antibodies and Lf-ANCA auto-antibodies and their concentration in umbilical cord blood serum (UCS) of CS-delivered newborns, verifying the sex-specificity of this relationship.

Firstly, however, we checked if the sex of newborns and the maternal concentration of examined antibodies were related to neonatal antibodies-related parameters. As previously observed^59–63^, the maternal concentration of IgG antibodies was negatively associated with the PTR and positively correlated with their concentration in the UCS. Additionally, our results seem to demonstrate that these relationships are sex independent. Similar correlation were observed for Lf-ANCA auto-antibodies, however there are no data in the literature for comparison as, to the best of our knowledge, our results are the first in this area.

In this study, an excessive pBMI in the examined women seems to impair the placental transport of IgG antibodies and significantly decreases their concentration in the UCS, which indicates a reduction in the immune defence potential of CS-delivered newborns. This phenomenon particularly applied to male newborns (Tables 5 and 4). Moreover, an excessive pBMI seems to be a stronger – than GWG – predictor of decreased PTR of IgG antibodies as its lowest value was demonstrated in newborns born to mothers with both excessive pBMI and GWG, whereas no significant differences in PTR of IgG antibodies was observed between newborns born to mothers with adequate pBMI but with different GWG (adequate or excessive) (Fig. 5). This observation was also particularly shown in male newborns. Additionally, results of our study seem to suggest that in overweight and obese pregnant women, an excessive GWG may intensify an adverse immunological effect of lowering PTR of IgG and their concentration in the UCA as well as increasing the PTR of Lf-ANCA and their concentration in the UCS (Figs. 3 and 4).

These results support previous literature reports demonstrating a clearly negative impact of maternal overweight and obesity on the immune functions of newborns. Wilson et al. (2015) found, in the UCS of newborns born to obese mothers, a reduced number and reactivity of specific (CD4 + Th) and non-specific (monocytes, eosinophils, dendritic) cells^44^. Moreover, Cifuentes-Zuniga et al. (2017) showed that cord blood macrophages from newborns of obese mothers have a basic anti-inflammatory phenotype, which may impair induction of the inflammation necessary to fight infection. Additionally, cord blood monocytes of these newborns showed ex vivo impaired functions – decreased production of pro- and anti-inflammatory cytokines and chemokines and reduced ability of these cells to migrate in response to stimulation with lipopolisaccharide^45^.

The weakened immune defence mechanisms of newborns born to obese mothers is also evidenced by a significantly increased risk of sepsis and necrotic enteritis, requiring treatment in an intensive care unit, compared to newborns of non-obese mothers^47,48^. Importantly, reduction of the immune defence potential of the offspring may apply not only in the neonatal period but also in further life. Griffiths et al. and Haberg et al. demonstrated an increased susceptibility of adults born to obese mothers to respiratory system infections^49,50^.

Studies carried out on an animal model also indicate impairment of immune functions in the offspring of obese mothers. Increased morbidity and mortality due to bacterial infections have been demonstrated in the offspring of obese female rats^51^, whereas in the peripheral blood cells of young baboons born to obese females, significant changes in the expression of genes involved in antigen presentation, the complement cascade and leucocyte migration were observed^52^.

The impairment of placental transport of IgG antibodies (and to a lesser extent – increase of PTR of Lf-ANCA) in pregnant women with pre-conception excess body weight – intensified by excessive GWG, demonstrated in the present study, may be explained by the probably limited expression of genes encoding neonatal Fc receptor (FcRn)^64^ as epigenetic modifications are considered to be the key mechanism linking maternal obesity with health outcomes in the offspring^65^. For example, in animal models, maternal obesity was demonstrated to up-regulate the expression of genes encoding placental transporters of nutrients, resulting in fetal overgrowth^66,67^ with adverse health consequences in later life^65^. However, the latest study suggested no significant correlation between the placental level of FcRn and the maternal to fetal transport rate of IgG antibodies^59^, which entails the need to search for an explanation of the mechanism of this relationship.

Some maternal factors limiting the effective FcRn-dependent placental transport of IgG antibodies to the fetus have been described, such as malnutrition, diabetes, hyperglycaemia, chronic infections (including malaria and HIV) and hypergammaglobulinaemia^43^. Additionally, the results of the present study may suggest that pre-conception overweight or obesity of pregnant women contributes to the limitation of placental transport of IgG antibodies and a significant reduction of their concentration in the UCS of CS-delivered newborns. Further studies, involving newborns from multiple pregnancies and natural births, are needed to explain if this potential risk factor is universal for the whole population of newborns or specific only for CS-delivered newborns.

Interestingly, this phenomenon particularly concerned male newborns, which seems to support the widespread view that male fetuses are more eco-sensitive and that adverse health effects are often more pronounced in male offspring^68,69^. Sexual dimorphism in terms of fetal programming (including immune programming) has not been unequivocally explained, although a different, sex-dependent, sensitivity of the organism to the same unfavourable factor (stressor) during prenatal development is commonly observed by researchers^68,69^. Similarly, the placentas of male and female fetuses show different sensitivity to the same stressor^68^ due to being the same sex (i.e. having XX or XY chromosomes) as the developing fetus^70^. It has even been suggested that male and female sex should be treated as two separate research models of fetal programming^69^.

In contrast to pBMI, GWG does not seem to be so pronounced associated with the PTR of IgG antibodies and Lf-ANCA auto-antibodies and their concentration in the UCS, despite the fact that both excessive BMI and excessive GWG promote low systemic inflammation and oxidative stress^71,72^, which can contribute to epigenetic modifications in expression of genes encoding FcRn^64,65^ as well as general impairment of immune health^73^. In our study, excessive GWG seems to be rather an additional factor – than an independent trigger – that may intensify an adverse immune effect, especially in overweight and obese women. Studies have shown that an excessive weight gain is significantly more common in women with preconceptional overweight and obesity^74,75^ that was also observed in our study – all women with an excessive pBMI was simultaneously characterized by an excessive GWG. Moreover, clinical trials have shown the lack of effectiveness of dietary interventions in overweight and obese pregnant women in reducing their weight during pregnancy^76^. This suggest that the problem of overweight and obesity in the preconception period is most often aggravated during pregnancy by excessive GWG which may have adverse immune consequences for the offspring. This suggestion seem to be supported by the results of our study which demonstrated that newborns delivered by CS to mothers with an excessive pBMI and GWG had the lowest PTR of IgG antibodies and their concentration in the UCS as well as the highest PTR of Lf-ANCA auto-antibodies and their concentration in the UCS.

## Conclusions

The results of the study seem to demonstrate that excessive pBMI appears to reduce the PTR of IgG antibodies and their concentration in the UCS of CS-delivered newborns, especially in male newborns. Moreover, an excessive GWG seems to play a role of an additional factor that commonly accompanies maternal overweight and obesity and may decrease PTR of beneficial IgG antibodies and increase PTR of detrimental Lf-ANCA auto-antibodies potentially leading to the impairment of neonatal immunity.

It should be emphasized that this is a pilot research, therefore, obtained results must be reconfirmed in further study. Exclusive participation of women with CS, no multivariate analyses (no conditions), no adjustment for confounding factors, small sample size, especially in sex-specific analyses, low proportion of women with obesity and undernutrition as well as low proportion of women with insufficient GWG, are the main limitations of this study. Therefore, it would be recommended:

- to conduct further study on a larger population of pregnant women and their newborn children that exceeds the minimum sample size (estimated minimum sample size is 380);
- to include equal proportion of women from all categories of pBMI (undernutrition, normal weight, overweight, obesity) as well as from all categories of GWG (insufficient, adequate and excessive);
- to use high-power statistical tests and multivariable model adjusted for confounding factors in order to confirm an independent influence of maternal pre-conception overweight and obesity on the PTR of examined antibodies and their concentration in the cord blood;
- to enroll into the study women with both CS and vaginal deliveries as well as with multiple pregnancies in order to generalize conclusions into whole population of pregnant women.

Given that maternal antibodies pass to the fetus via the placenta during pregnancy, with the highest rate within the 4 last weeks (37–41 Hbd)^53^, at the time of delivery, the child has as many antibodies as it received in the prenatal period. This seems to suggest that the mode of delivery (vaginal vs. CS) shouldn’t affect the cord blood antibodies concentration at birth, and the exclusion of women with natural deliveries shouldn’t affect the generalizability of conclusions to the entire population of pregnant women. However, as it was not demonstarated in our study or in another studies, the exclusion of women with natural birth was considered as a methodological limitation. Additionally, further full-scale studies on this issue could:

- include extended analysis of the nutritional status of women in the pre-conception period, including body composition or protein, fat and mineral nutritional status;
- include more indicators of the immunological defence potential of the offspring, such as number and activity of various types of white blood cells, efficiency of complement system or level of secretory antibodies on the surface of the mucosa;
- include longitudinal observations of the immunological health of the offspring in order to explain the long-term effects.

Despite the limitations, results obtained in our pilot study seem to be a good starting point to further investigations as they demonstrated that maternal pre-conception overweight/obesity may negatively affect immunological defence potential of newborns and excessive GWG could intensify this adverse effect. Further extensive, longitudinal research on this issue, conducted on a sufficiently large study group and adjusted for potential confounding factors, will guarantee the scientific credibility of the results, enable generalization of the results from the sample to the entire population and enable application of the results to create nutritional recommendations for women in the pre-pregnancy period aimed at improving the immunological health of newborns and later in life.

## Electronic supplementary material

Below is the link to the electronic supplementary material.

Supplementary Material 1

## Data Availability

Data are available on reasonable request from the corresponding author.
