# Supplementary material for: Sex-specific association of immunological markers in CS-delivered newborns with pre-pregnancy body mass index and gestational weight gain of mothers
Source: Sci Rep. 2025 Jan 24;15:3074. doi: 10.1038/s41598-025-85711-x (PMC11760347; doi:10.1038/s41598-025-85711-x)
Supplement: Supplementary file 1 — Supplementary Material 1 [file 41598_2025_85711_MOESM1_ESM.pdf]

Sex-specific association of immunological markers in CS-delivered newborns with pre-pregnancy body mass index and gestational weight gain of mothers

Karolina Rak [1]*, Michaela Godyla-Jabłoński[1] and Monika Bronkowska [2]

<u>IgG antibodies determination</u>

- Material: umbilical cord blood serum of newborns (UCS IgG), maternal blood serum (MS IgG)
- Method: in duplicate; the immunoenzymatic method (ELISA); Human IgG Total ELISA Ready-SET-Go! kits (eBioscience Bender MedSystems GmbH; catalog number 88-50550). In two steps: 1) Coating of the plates 2) the actual determination - according to the procedure recommended by the manufacturer and included in the user manual.
- Absorbance measurement: Epoch microspectrophotometer (Bio-tek Instruments, USA).
- Units: IgG concentrations in ng/ml were converted and expressed in mg/dl.
- Calculation of placental transport rate (PTR): the ratio of UCS IgG to MS IgG (UCS/MS IgG).

After preparing the reagents according to the manufacturer's instructions, the plates were 1) coated with capture antibodies according to the scheme presented in Figure 1, and then 2) IgG antibodies in blood serum were determined according to the scheme presented in Fig. 2.

<u>Lf-ANCA auto-antibodies determination</u>

- Material: umbilical cord blood serum of newborns (UCS Lf-ANCA), maternal blood serum (MS Lf-ANCA)
- Method: in duplicate; the immunoenzymatic method (ELISA); Lactoferrin Ab ELISA kits (Demeditec Diagnostics GmbH, catalog number DE-7160),according to the procedure recommended by the manufacturer and included in the user manual
- Absorbance measurement: Epoch microspectrophotometer (Bio-tek Instruments, USA).
- Units: Lf-ANCA concentrations in U/ml
- Calculation of placental transport rate (PTR): the ratio of UCS Lf-ANCA to MS Lf-ANCA (UCS/MS Lf-ANCA).

After preparing the reagents according to the manufacturer's instructions, Lf-ANCA auto-antibodies determined in blood serum according to the scheme presented in Fig. 3.

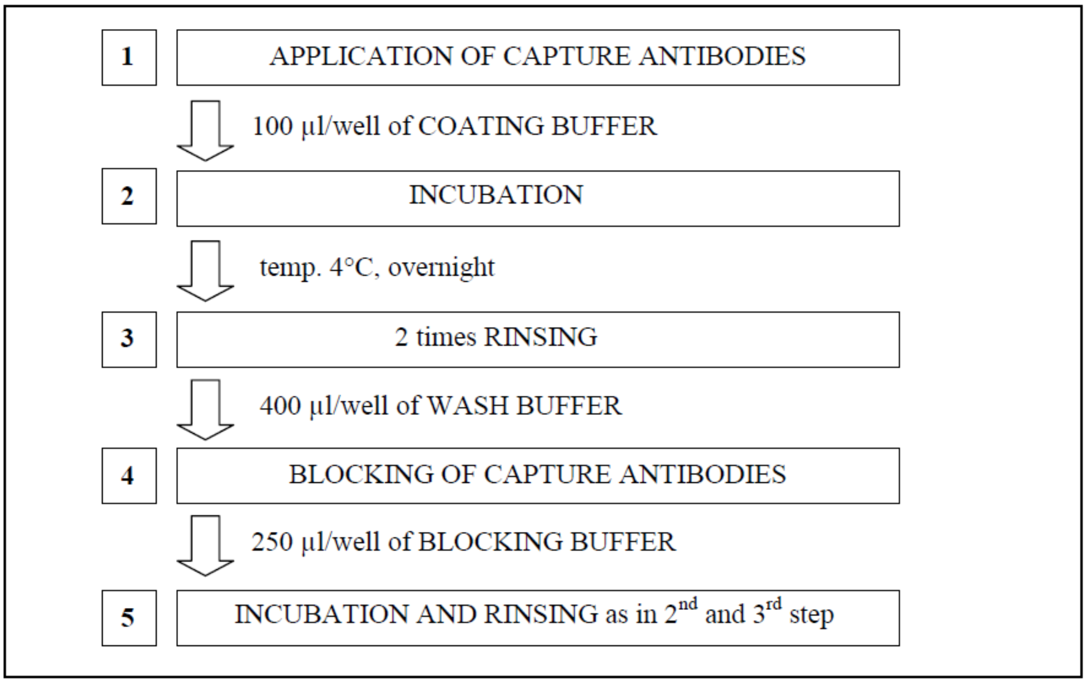

Fig. 1. Scheme of coating plates for the determination of IgG antibodies by ELISA method

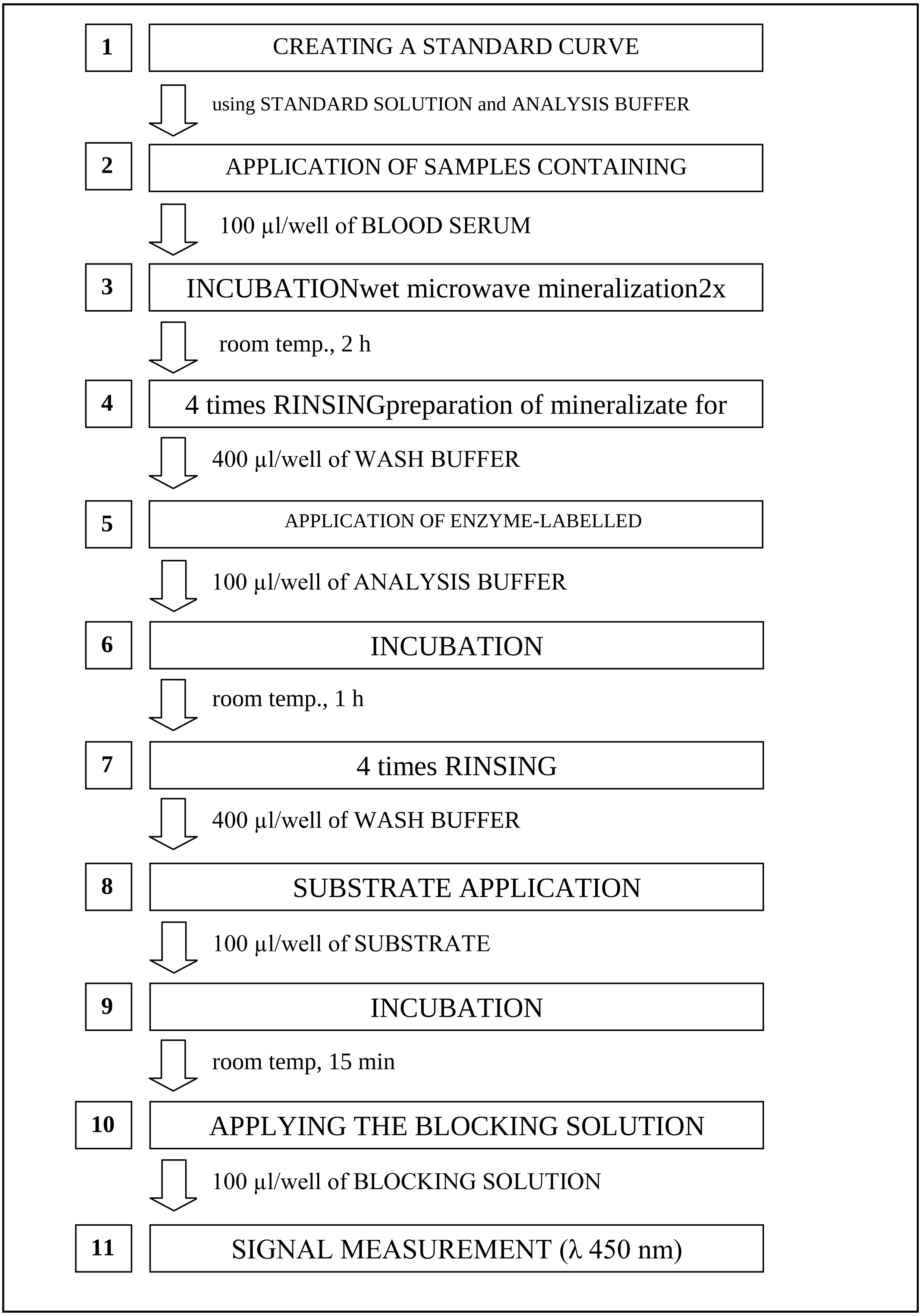

Fig. 2 Scheme of IgG antibody determination by ELISA method

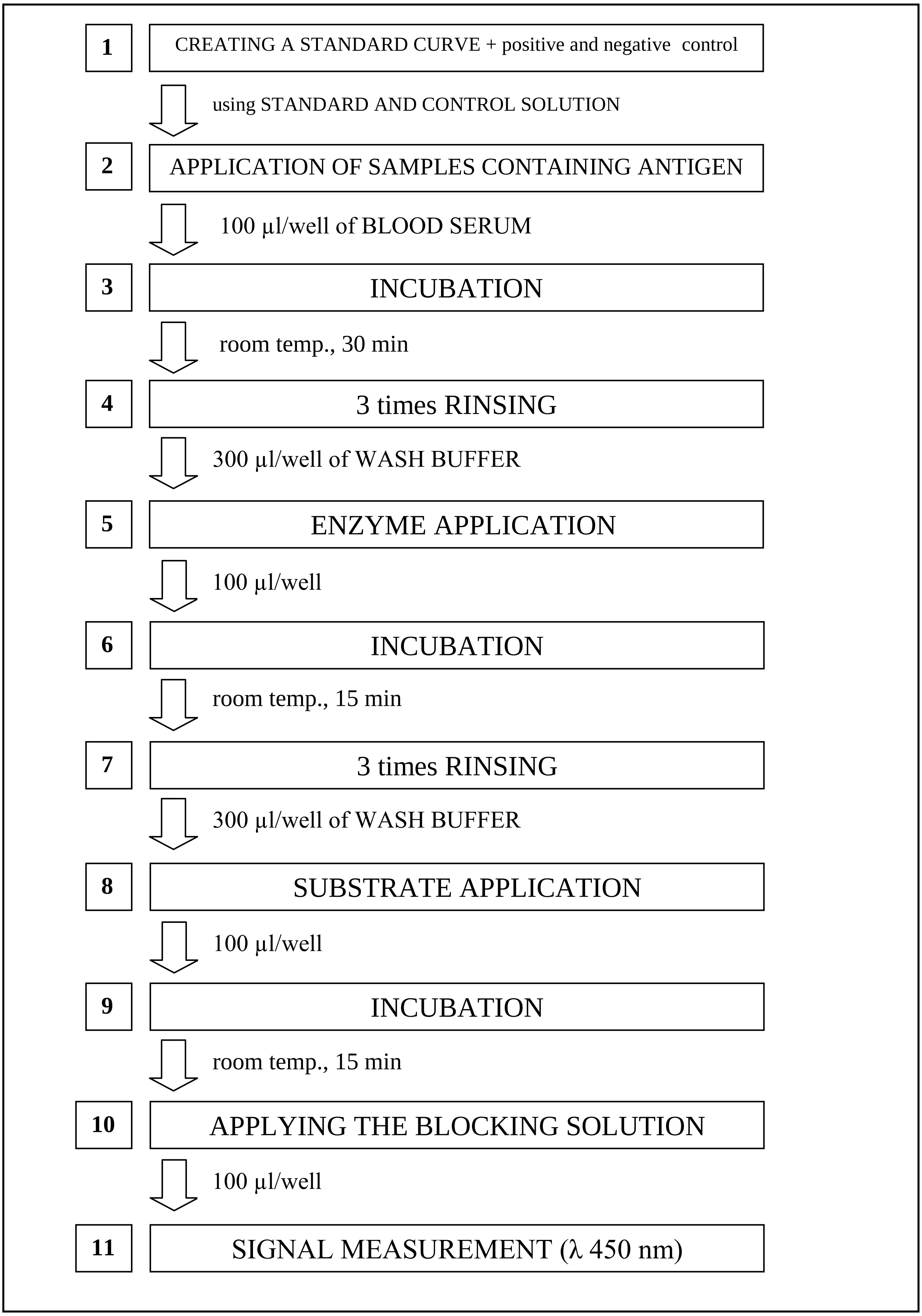

Fig. 3. Scheme of Lf-ANCA auto-antibody determination by ELISA method
